# Spheroid cancer stem cells display reprogrammed metabolism and obtain energy by actively running the tricarboxylic acid (TCA) cycle

**DOI:** 10.18632/oncotarget.8947

**Published:** 2016-04-23

**Authors:** Masakazu Sato, Kei Kawana, Katsuyuki Adachi, Asaha Fujimoto, Mitsuyo Yoshida, Hiroe Nakamura, Haruka Nishida, Tomoko Inoue, Ayumi Taguchi, Juri Takahashi, Satoko Eguchi, Aki Yamashita, Kensuke Tomio, Osamu Wada-Hiraike, Katsutoshi Oda, Takeshi Nagamatsu, Yutaka Osuga, Tomoyuki Fujii

**Affiliations:** ^1^ Department of Obstetrics and Gynecology, Graduate School of Medicine, The University of Tokyo, Bunkyo-ku, Tokyo, Japan

**Keywords:** cancer stem cell (CSC), metabolomics, ovarian cancer, cervical cancer, tricarboxylic acid (TCA) cycle

## Abstract

The Warburg effect is a metabolic hallmark of cancer cells; cancer cells, unlike normal cells, exclusively activate glycolysis, even in the presence of enough oxygen. On the other hand, intratumoral heterogeneity is currently of interest in cancer research, including that involving cancer stem cells (CSCs). In the present study, we attempted to gain an understanding of metabolism in CSCs that is distinct from that in non-CSCs. After forming spheroids from the OVTOKO (ovarian clear cell adenocarcinoma) and SiHa (cervical squamous cell carcinoma) cell lines, the metabolites of these cells were compared with the metabolites of cancer cells that were cultured in adherent plates. A principle components analysis clearly divided their metabolic features. Amino acids that participate in tricarboxylic acid (TCA) cycle reactions, such as serine and glutamine, were significantly increased in the spheroids. Indeed, spheroids from each cell line contained more total adenylates than did their corresponding cells in adherent cultures. This study demonstrated that cancer metabolism is not limited to aerobic glycolysis (i.e. the Warburg effect), but is flexible and context-dependent. In addition, activation of TCA cycles was suggested to be a metabolic feature of CSCs that was distinct from non-CSCs. The amino acid metabolic pathways discussed here are already considered as targets for cancer therapy, and they are additionally proposed as potential targets for CSC treatment.

## INTRODUCTION

The Warburg effect, or aerobic glycolysis, is a metabolic hallmark of cancer cells [1]. Normal cells activate the tricarboxylic acid (TCA) cycle to efficiently obtain energy or ATP in the presence of adequate oxygen; however, cancer cells exclusively activate glycolysis, even in the presence of enough oxygen.

On the other hand, it is well-documented that tumors have phenotypic and functional heterogeneity [2–4]. In light of this heterogeneity, many researchers are now paying attention to cancer stem cells (CSCs) [5].

Taken together, we became interested in the heterogeneity in cancer cells in terms of metabolism. In particular, we were interested in the Warburg effect because its employment has been posited to differ in the metabolism of cancer cells vs non-cancerous cells. In the present study, we attempted to gain an understanding the characteristics of metabolism in CSCs that may be distinct from that in non-CSCs.

One of the experimental approaches for obtaining CSC-like properties in cells is to culture cancer cells in suspension, resulting in spheroid-shaped cells [6–8]. Indeed, a previous study has suggested that metabolic profiles in spheroid-derived cells from ovarian serous adenocarcinoma were different from those in cancer cells that were cultured in adherent plates [8]. The significance and implications of this difference were not fully discussed.

This study aimed to gain insight into universal CSC metabolic processes by using OVTOKO (ovarian clear cell adenocarcinoma) and SiHa (human papilloma virus-16 positive cervical squamous cell carcinoma) cell lines. The cancer cells were cultured in adherent plates (described later as 2-dimensional or 2D) and in low-attachment plates (described later as 3-dimensional or 3D), and a metabolome analysis was performed to detect differences in metabolites between OVTOKO-2D or -3D and SiHa-2D or -3D conditions.

A principal components analysis clearly divided these four groups. Additionally, the differences in the metabolites generated under 3D and 2D conditions were attributable to the amino acids that are essential for actively and efficiently carrying out TCA cycle reactions; namely, serine, aspartate, glutamate and glutamine [6, 9–15]. Indeed, these amino acids were significantly increased in 3D conditions when compared to 2D conditions. Accordingly, OVTOKO-3D and SiHa-3D cells contained more total adenylates than did OVTOKO-2D and SiHa-2D cells, respectively. We herein report that activation of TCA cycles appears to be a metabolic feature of CSCs that distinguishes them from non-CSCs. Although metabolic pathways of the amino acids, especially serine and glutamine, are already considered as targets for cancer therapy [6, 14, 15], they are additionally proposed as potential targets for CSC treatment.

## RESULTS

### OVTOKO-3D and SiHa-3D cells express CSC marker(s) more highly than do OVTOKO-2D and SiHa 2D cells, respectively

**Figure 1:**
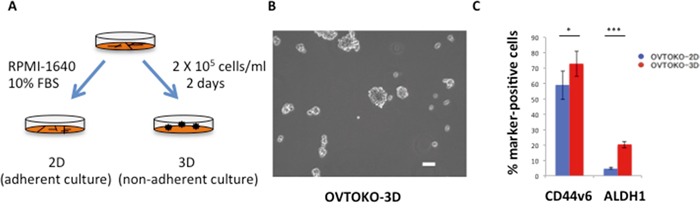
Outline of the present study **A.** Schema of experimental procedures RPMI-1640 medium was used for these experiments. Spheroid medium, which contains epidermal growth factor (EGF), fibroblast growth factor-2(b-FGF) and a variety of other supplements, was not used to eliminate the influence of these supplements on metabolism. **B.** Representative image of a spheroid Culturing cancer cells in low-attachment plates produced spheroids. Bar = 100 μm. **C.** Percentage of cancer stem cell marker-positive cells. OVTOKO-3D cells expressed CSC markers significantly more than OVTOKO-2D cells. Experiments were repeated at least three times. The values shown represent the means ± S.Ds. ***, p < 0.001; *, p < 0.05.

The presence of a population of CSC marker-positive cells was confirmed in spheroids. The experimental procedures and a representative image of a spheroid are shown in Figures 1A and 1B. Although a CSC marker for ovarian clear cell adenocarcinoma is yet to be identified, CD44v6 and aldehyde dehydrogenase 1 (ALDH1) activities are considered in the literature as candidates for CSC markers [16, 17]. Indeed, OVTOKO-3D cells expressed CSC markers more highly than did OVTOKO-2D cells (Figure 1C). Likewise, SiHa-3D cells expressed greater ALDH1 (which has been reported as a cervical CSC marker) activity than did SiHa-2D cells, as was previously reported (data not shown) [18].

### OVTOKO-3D and SiHa-3D cells have lower levels of intracellular reactive oxidative species (ROS) stress compared with OVTOKO-2D and SiHa-2D cells, respectively

**Figure 2:**
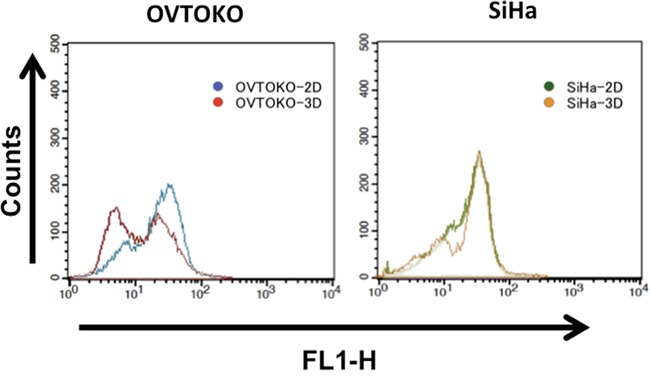
Intracellular reactive oxidative species (ROS) activities in 3D and 2D conditions OVTOKO-3D and SiHa-3D cell ROS activities showed binomial distribution patterns. These patterns suggest that the spheroids are hybrids, at least in terms of metabolism.

As is shown in Figure 2, OVTOKO-3D and SiHa-3D cells showed binomial distribution patterns of ROS activities, which suggested that there was metabolic heterogeneity within these cell cultures. It was also suggested that cancer cells in 3D conditions had lower levels of ROS stress compared to cancer cells in 2D conditions.

### Principal components analysis (PCA) distinguishes the metabolic profiles of OVTOKO-2D, 3D and SiHa-2D, 3D

**Figure 3:**
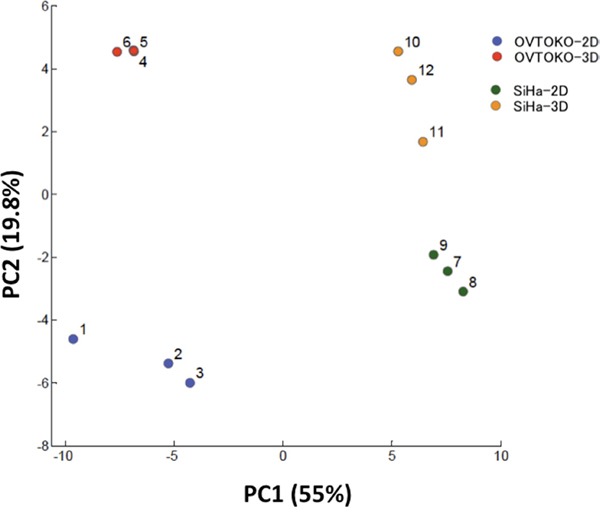
Principal components analysis (PCA) for metabolome analysis The PCA completely separated the metabolic profiles of four groups (n=3 in each group). Principal component (PC) 1 captured the cell type separation; PC 2 captured differences inculture conditions (2D vs 3D).

As is shown in Figure 3, PCA completely separated the metabolic profiles of the four groups. Principal component (PC) 1 was interpreted as capturing the cell type separation and PC 2 was interpreted as capturing the culture condition separation (2D vs 3D). The aim of the present study was to gain insight into the metabolic features of CSCs that are distinct from non-CSCs, and thus the major factors of PC2 became the focus of this study.

### Amino acids that are advantageous to the tricarboxylic acid (TCA) cycle are the top metabolites in PC2

**Figure 4:**
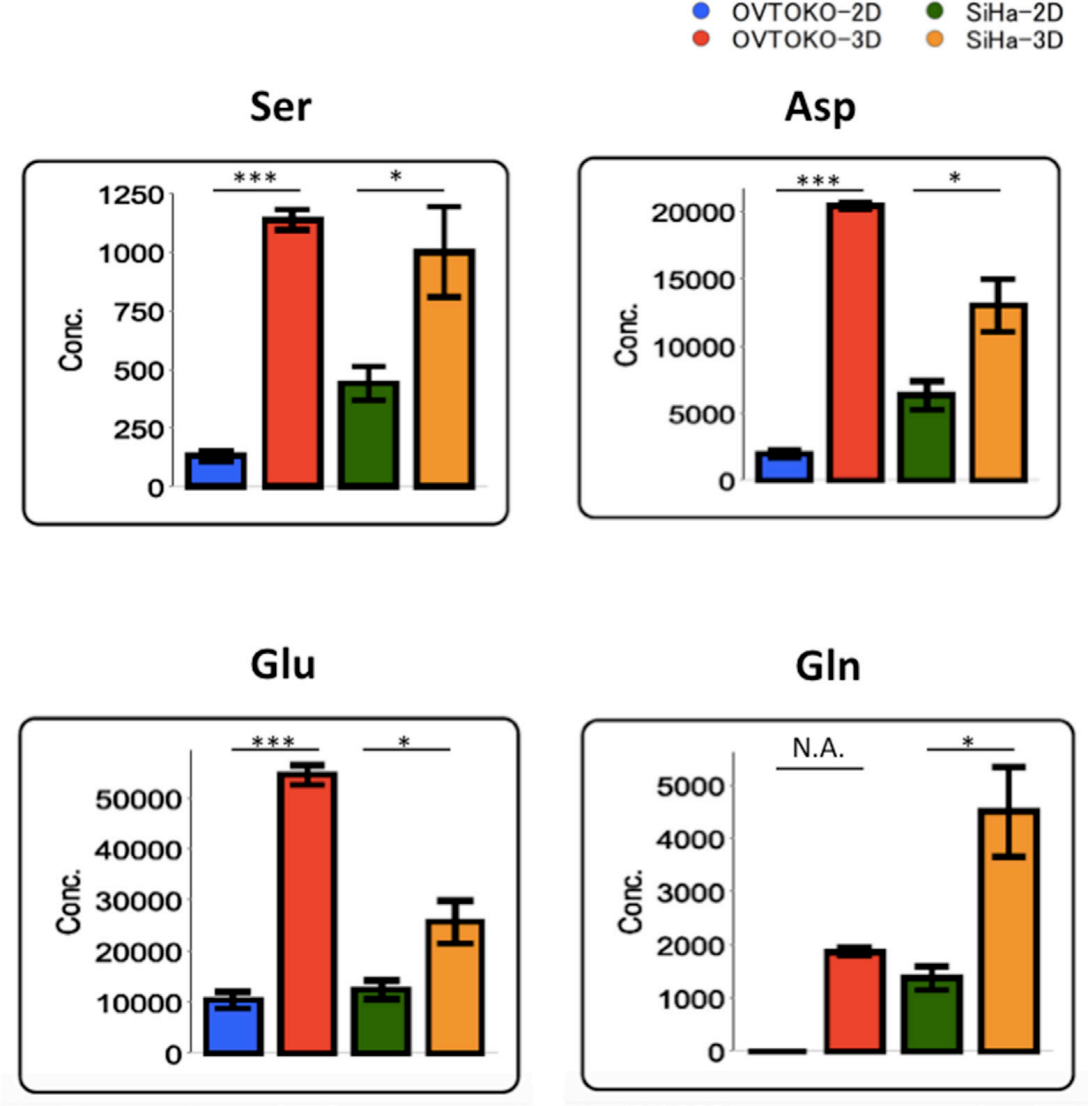
Specific amino acids with the largest separation in PC2 factor loadings Amino acid concentrations are markedly increased in 3D conditions relative to 2D conditions. The values shown represent the mean ± S.D. ***, p < 0.001; *, p < 0.05; N.A., not available.

Factor loadings for PC2 are shown in Table 1. Serine, aspartate, glutamate and glutamine are the top amino acids that accounted for PC2 separation. Indeed, these metabolites are significantly higher in the 3D group than in the 2D group (Figure 4).

**Table 1:** Top and bottom 30 metabolites in PC2 factor loadings

Metabolites (Top 30)	Factor loadings	Metabolites (Bottom 30)	Factor loadings
Ser	2.3E-01	…	…
Asp	2.2E-01	Asn	−3.2E-02
Glu	2.0E-01	Xanthine	−3.4E-02
Gln	1.7E-01	Cystathionine	−4.0E-02
Creatine	1.7E-01	Ribose 1-phosphate	−4.1E-02
*N*-Carbamoylaspartic acid	1.6E-01	Ala	−4.9E-02
NADH	1.5E-01	2-Oxoisovaleric acid	−5.1E-02
Gly	1.5E-01	2-Phosphoglyceric acid	−5.4E-02
Succinic acid	1.5E-01	Hydroxyproline	−6.7E-02
Carnitine	1.4E-01	Adenosine	−7.1E-02
Glutathione (GSH)	1.4E-01	Citrulline	−7.6E-02
GDP	1.4E-01	Carnosine	−9.1E-02
Glutathione (GSSG)	1.4E-01	3-Phosphoglyceric acid	−9.3E-02
NADPH	1.4E-01	cAMP	−9.6E-02
Malic acid	1.4E-01	Lys	−9.6E-02
cGMP	1.3E-01	Lactic acid	−9.7E-02
Citric acid	1.3E-01	Galactose 1-phosphate	−1.0E-01
ADP	1.3E-01	IMP	−1.1E-01
GMP	1.2E-01	Glucose 1-phosphate	−1.2E-01
Argininosuccinic acid	1.2E-01	Pro	−1.2E-01
Fumaric acid	1.2E-01	PRPP	−1.2E-01
γ-Aminobutyric acid	1.1E-01	Acetyl CoA	−1.3E-01
AMP	1.0E-01	Urea	−1.3E-01
2-Hydroxyglutaric acid	1.0E-01	Arg	−1.3E-01
Adenylosuccinic acid	1.0E-01	Inosine	−1.3E-01
2-Oxoglutaric acid	1.0E-01	Phosphoenolpyruvic acid	−1.3E-01
NADP*	1.0E-01	Dihydroxyacetone phosphate	−1.4E-01
CoA	9.4E-02	Ornithine	−1.5E-01
Malonyl CoA	9.4E-02	Glyceraldehyde 3-phosphate	−1.6E-01
*cis*-Aconitic acid	9.3E-02	XMP	−1.7E-01
…	…	Ribose 5-phosphate	−1.7E-01

The top 30 metabolites include specific amino acids that are used for efficiently carrying out TCA cycle reactions, TCA cycle intermediates and glutathione. The bottom 30 metabolites include glycolysis and PPP intermediates.

PC, principal component; TCA, tricarboxylic acid; PPP, pentose phosphate pathway.

### Summary of the metabolomic profiles shows drastic differences in reprogramming of metabolism between 3D and 2D conditions

**Figure 5:**
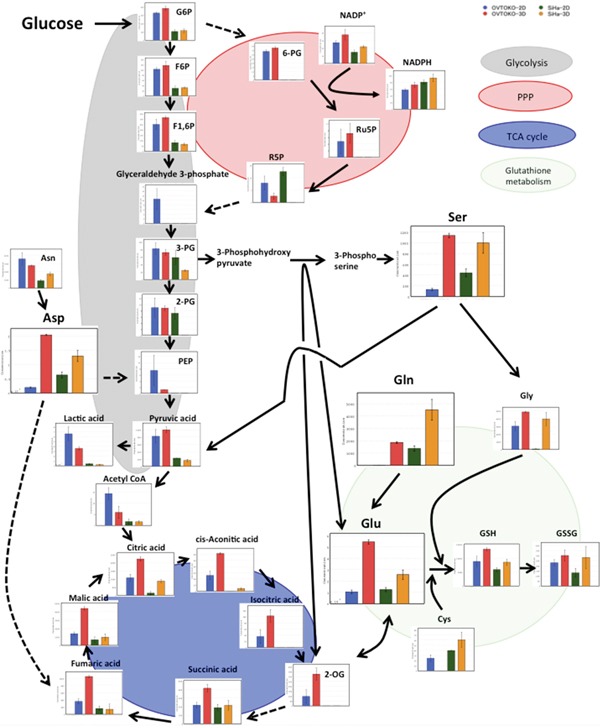
Summary of the metabolomic profiles in 2D vs. 3D cultures for each cell line 3D metabolism was drastically reprogrammed compared to 2D metabolism. 3D metabolism patterns suggest an increased contribution of the TCA cycle when compared to 2D metabolism. The values shown represent the means ± S.Ds. The lack of a bar graph representation for a given metabolite means that the metabolite was not detected. Solid lines represent neighboring reactions, and dashed lines represent that some reactions were omitted due to space limitations.

**Figure 6:**
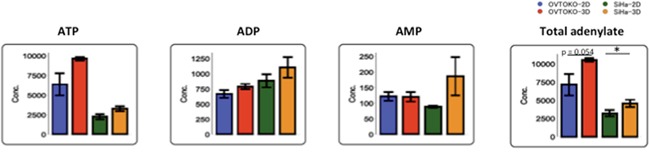
Changes in adenylates according to culture conditions Total adenylates were increased in 3D conditions compared to 2D conditions. The values shown represent the means ± S.Ds. *, p < 0.05.

A summary of the changes in metabolites is shown in Figure 5. Lactic acid, which is the final metabolite in glycolysis, was present in lower concentrations in 3D conditions than in 2D conditions. Meanwhile, citric acid, which is the first metabolite in the TCA cycle, was present in higher concentrations in 3D conditions than in 2D conditions. These results suggested that the TCA cycle is more active in OVTOKO-3D and SiHa-3D cells than in OVTOKO-2D and SiHa-2D cells, respectively. Indeed, total adenylates were present in higher concentrations in OVTOKO-3D and SiHa-3D cells than in OVTOKO-2D and SiHa-2D cells, respectively (Figure 6).

## DISCUSSION

This study investigated metabolic features of cancer stem cells (CSCs) that were distinct from non-CSCs in gynecological malignancies.

Aerobic glycolysis, which is known as the Warburg effect, is thought to be a hallmark of cancer cells [1]. On the other hand, intratumoral heterogeneity, which results in resistance to cancer treatment, is now a common research focus [2–4]. In particular, CSCs are currently being investigated in various types of solid tumors [5].

In the present study, a metabolome analysis demonstrated that CSC-like cells carried out the TCA cycle more actively than non-CSCs.

First, cancer cells were cultured in suspension (described as -3D, while normal adherent culture is described as -2D), which formed spheroids, as an experimental approach to obtain CSC-like properties. Indeed, OVTOKO (ovarian clear cell adenocarcinoma)-3D and SiHa (human papilloma virus-16 positive cervical squamous cell carcinoma)-3D cells express CSC marker(s) more highly than do OVTOKO-2D and SiHa-2D cells, respectively, as previously described [16–18].

Secondly, a metabolome analysis was performed using these cancer cells. A principal component analysis (PCA) clearly divided the metabolic patterns of these cells into four groups. The factors that account for metabolic changes in 3D conditions when compared to 2D conditions were the amino acids that participate in the TCA cycle (Table 1 and Figure 5). These amino acids were significantly increased under 3D conditions when compared to 2D conditions, which suggested that cancer cells in 3D conditions activate the TCA cycle. Accordingly, the production of lactic acid, which is the final metabolite in glycolysis, was lower in 3D than in 2D conditions. Citric acid, which is the first metabolite in the TCA cycle, was present in higher concentrations in 3D than in 2D conditions. The total adenylates in 3D conditions are greater than in 2D conditions (Figure 6). Taken together, these results suggest that OVTOKO-3D and SiHa-3D cells use the TCA cycle more actively than do OVTOKO-2D and SiHa-2D cells. Therefore, activation of TCA cycles may be a metabolic features in CSCs that distinguish them from non-CSCs.

Most commonly, medium for spheroid culture contains epidermal growth factor (EGF), fibroblast growth factor-2 (b-FGF) and a few other supplements [8, 18]. However, the use of this medium introduces challenges in the interpretation of metabolome results because the metabolites that are influenced by these supplements cannot easily be distinguished from those produced endogenously. For instance, EGF signaling is known to activate glycolysis [19]. For this reason, we chose RPMI-1640 medium for suspension cultures. The experimental procedures that were used in this study enabled a focus on the metabolism influenced only by the presence or absence of a particular scaffolding.

One limitation of this study is that we used only two cell lines. However, we specifically chose cell lines that differed in terms of origin (ovary or cervix) and pathology (adenocarcinoma or squamous cell carcinoma). Still, because the cell lines differed, we limited our conclusions to the factors associated with PC2, which we felt would be independent of cancer cell type and could therefore be potential targets for CSC treatment.

Although differences in metabolites between the cell types (PC1) were not the focus of this study, it would be of interest to delve further into this data because this factor could reflect differences in clinical behaviour between ovarian clear cell carcinomas and cervical squamous cell carcinomas. For instance, the concentrations of total amino acids were significantly higher in 3D conditions than in 2D conditions for both OVTOKO and SiHa cells, however, the attributes between them were completely different; almost all kinds of amino acids were increased in SiHa-3D compared to SiHa-2D, on the other hand, a much more limited number of amino acids contributed to the increase in total amino acids in OVTOKO cells (Figure S1A and S1B).

In conclusion, the activation of the TCA cycle was here shown to be a specific metabolic feature of CSCs but not of non-CSCs. Metabolic pathways involving the amino acids discussed here, especially serine and glutamine, are already under consideration as targets for cancer therapy [12, 14, 15, 20],; we propose they are also potential targets for CSC treatment. It is not yet clear whether this reprogramming is caused by changes in enzymatic expression [9, 13, 21], in the expression of transporters [22], in amino acid sensors or in morphology [7, 22–24]. The discovery of the key mechinisms behind metabolic reprogramming in CSC is an important goal for future investigative efforts.

## MATERIALS AND METHODS

### Cell lines and cell culture

The cancer cell lines OVTOKO and SiHa were obtained from American Type Culture Collection (ATCC, USA) and cultured in RPMI-1640 medium (Wako, Japan. Lot # ECH7023) supplemented with 10% fetal bovine serum (FBS; Invitrogen, USA. Lot# 1606672) and 100 U/ml penicillin/ 100 μg/ml streptomycin(Wako), and sub-cultured by 0.25% trypsin/EDTA (Wako) detachment. All cells were grown in a humidified atmosphere at 37°C and 5% CO_2_. Media and FBS from the same lot were used for all experiments (including suspension cultures).

### Suspension (spheroid-forming) culture

Dissociated single cells (2 × 10^5^ cells/ml) were seeded into ultra-low attachment plates (Corning, USA) and were cultured for 2 days. For collecting spheroids, medium was centrifuged for 2 min at 100 X g and the supernatants were carefully aspirated. Prior to each experimental procedure (except metabolome analysis), collected spheroids were dissociated with Accutase (Gibco, USA).

### Flow cytometry

Flow cytometric analysis was performed as described [17]. 1×10^6^ OVTOKO cells were stained with anti-CD44v6 antibodies (R&D systems, USA) according to the manufacturer's instructions and sorted using a FACS Calibur flow cytometer (BD Biosciences, Japan). The ALDH enzymatic activity of OVTOKO and SiHa cells (1×10^6^ cells) was measured as described [18], using the ALDEFLUOR kit (STEMCELL Technologies, Canada). Experiments were repeated at least three times.

### Intracellular reactive oxygen species (ROS) assays

Dissotiated single cells were incubated with 2′,7′-dichlorodihydrofluorescein diacetate (DCF-DA, Sigma-Aldrich, USA) at 37°C for 40 min. After washing, cells were exposed to 100 mM H_2_O_2_ (Wako) for 20 min. Fluorescence intensity was measured using a FACS Calibur flow cytometer.

### Metabolite measurements

Metabolome analysis was carried out through a facility service(C-SCOPE) at Human Metabolome Technologies, Inc.(HMT, Inc., Japan) as previously described (n=3 in each group) [25, 26]. A total of 116 metabolites were analyzed. When collecting samples, adherent culture cells were removed using a cell scraper in an effort to exclude the possible influence of the dissociation solution. They were subsequently treated with protocols identical to those employed with spheroids. Washes were performed using 5% mannitol.

### Statistical analysis

A two tailed t-test was used to compare the means of populations of CSC marker positive cells (flow cytometry). Welch's t-test was used to compare the means of metabolite concentrations in metabolome analysis. P values less than 0.05 were considered statistically significant. The JMP^®^/SAS Institute was used for statistical analysis. In metabolome analysis, SampleStat ver. 3.14 was used for statistical analysis (HMT).

## SUPPLEMENTARY FIGURE


