# Supplementary material for: Spheroid cancer stem cells display reprogrammed metabolism and obtain energy by actively running the tricarboxylic acid (TCA) cycle
Source: Oncotarget. 2016 Apr 23;7(22):33297–305. doi: 10.18632/oncotarget.8947 (PMC5078095; doi:10.18632/oncotarget.8947)
Supplement: Supplementary file 1 [file oncotarget-07-33297-s001.pdf]

Spheroid cancer stem cells display reprogrammed metabolism and obtain energy by actively running the tricarboxylic acid (TCA) cycle

SUPPLEMENTARY FIGURE

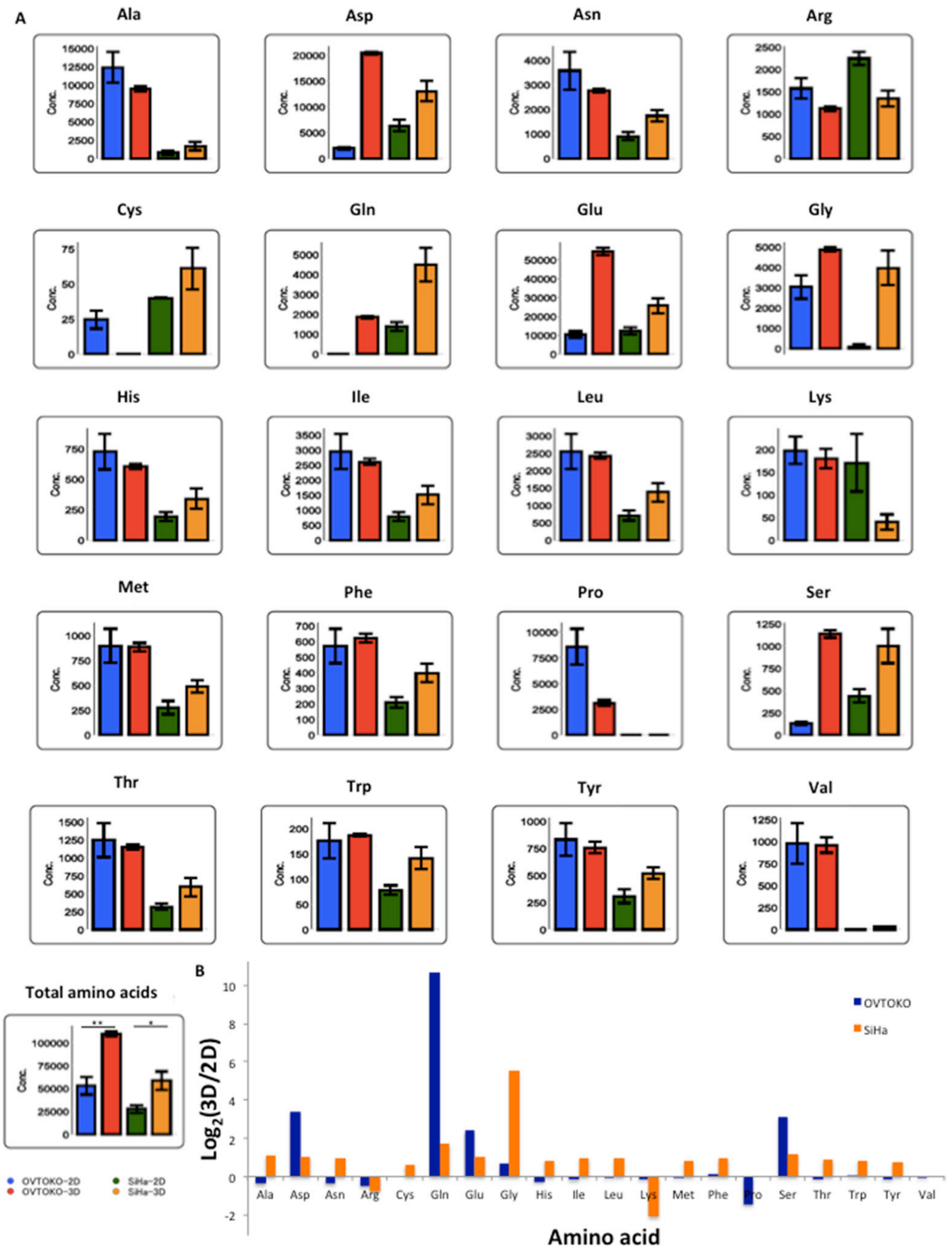

Supplementary Figure S1: Changes in amino acid concentrations in OVTOKO and SiHa cells. A. Changes in amino acid concentrations in OVTOKO and SiHa cells. Concentrations of total amino acids were increased in both cell lines. The values shown represent the means ± S.Ds. No bar graph indicates that the metabolite was not detected. **, $p < 0.01$; *, $p < 0.05$. **B.** Logarithmic transformations of the ratios of each amino acid concentration (3D/2D). The graph clearly shows that the patterns of the amino acid concentration changes differ dramatically between OVTOKO and SiHa cells.
